# Sputum Leucine-Rich Alpha-2 Glycoprotein as a Marker of Airway Inflammation in Asthma

**DOI:** 10.1371/journal.pone.0162672

**Published:** 2016-09-09

**Authors:** Hiromi Honda, Minoru Fujimoto, Shintaro Miyamoto, Nobuhisa Ishikawa, Satoshi Serada, Noboru Hattori, Shintaro Nomura, Nobuoki Kohno, Akihito Yokoyama, Tetsuji Naka

**Affiliations:** 1 Laboratory of Immune Signal, National Institutes of Biomedical Innovation, Health and Nutrition, Ibaraki, Osaka, Japan; 2 Department of Haematology and Respiratory Medicine, Kochi University, Nankoku-shi, Kochi, Japan; 3 Department of Respiratory Medicine, Hiroshima Prefectural Hospital, Minami-ku, Hiroshima, Japan; 4 Department of Molecular and Internal Medicine, Graduate School of Biomedical Science, Hiroshima University, Minami-ku, Hiroshima, Japan; 5 Department of Animal Bioscience, Nagahama Institute of Bio-Science and Technology, Nagahama, Shiga, Japan; 6 Division of Translational Research, Integrated Center for Advanced Medical Technologies (ICAM-Tech), Kochi Medical School, Kochi University, Nankoku-shi, Kochi, Japan; National and Kapodistrian University of Athens, GREECE

## Abstract

**Background:**

Asthma is a chronic inflammatory disease of airways, but an ideal biomarker that accurately reflects ongoing airway inflammation has not yet been established. The aim of this study was to examine the potential of sputum leucine-rich alpha-2 glycoprotein (LRG) as a new biomarker for airway inflammation in asthma.

**Methods:**

We obtained induced sputum samples from patients with asthma (N = 64) and healthy volunteers (N = 22) and measured LRG concentration by sandwich enzyme-linked immunosorbent assay (ELISA). Ovalbumin (OVA)-induced asthma model mice were used to investigate the mechanism of LRG production during airway inflammation. The LRG concentrations in the bronchoalveolar lavage fluid (BALF) obtained from mice were determined by ELISA and mouse lung sections were stained with anti-LRG antibody and periodic acid-Schiff (PAS) reagent.

**Results:**

Sputum LRG concentrations were significantly higher in patients with asthma than in healthy volunteers (p = 0.00686). Consistent with patients’ data, BALF LRG levels in asthma model mice were significantly higher than in control mice (p = 0.00013). Immunohistochemistry of lung sections from asthma model mice revealed that LRG was intensely expressed in a subpopulation of bronchial epithelial cells, which corresponded with PAS-positive mucus producing cells.

**Conclusion:**

These findings suggest that sputum LRG is a promising biomarker of local inflammation in asthma.

## Introduction

Asthma is a chronic inflammatory disease of the airways, characterized by bronchial hyper-reactivity, airway obstruction, and mucus hyper-production. Although pulmonary function tests are often used to objectively assess the severity of the disease, they do not necessarily reflect ongoing airway inflammation. Indeed, several biomarkers have been evaluated for sputum, bronchoalveolar lavage fluid (BALF), and exhaled samples in order to assess the inflammation levels of the airways as well as therapeutic effects of an intervention. For example, fractional exhaled nitric oxide (FeNO) is a widely used exhaled marker of airway inflammation, and is thought to be specific for eosinophilic inflammation in asthma patients [1]. However, recent evidence suggests that single measurements of FeNO are insufficient to evaluate asthma control and to determine anti-inflammatory medication dosing [2, 3]. The search for novel biomarkers of airway inflammation is warranted to establish accurate diagnosis, monitoring disease progression and personalizing treatment.

Leucine-rich alpha-2 glycoprotein (LRG) was identified as a serum protein containing eight leucine-rich repeats [4, 5]. LRG expression is up-regulated in granulocytes during their differentiation [6] and in hepatocytes during the acute phase response [7]. We have previously reported that serum LRG is a disease activity marker for inflammatory diseases such as rheumatoid arthritis and ulcerative colitis [8, 9]. Given that inflamed tissues can produce LRG [9], it seemed logical that LRG concentrations in samples collected from the site of inflammation might reflect the severity of local inflammation. Therefore, in this study, we investigated the significance of sputum LRG as a novel biomarker of ongoing airway inflammation in asthma.

## Materials and Methods

### Study Subject

We obtained induced sputum samples from patients diagnosed with bronchial asthma (N = 64) and healthy volunteers without respiratory symptoms (N = 22). The collection of induced sputa was approved by the ethics committee of Hiroshima University, and all subjects provided written, informed consent. Sputum specimens were obtained and processed as previously described by using dithiothreitol (DTT) [10–12]. The clinical characteristics of the study subjects are shown in Table 1. Individual data sets of patients’ characteristics are provided in S1 File.

**Table 1:** Clinical characteristics of the study subjects.

	Asthma	HV
**Subjects**	64	22
**Females/Males**	35/29	14/8
**Age years**	55.6 ± 15.4	58.4 ± 10.4
**Disease duration (y)**	17.1 ± 12.7	-
**Smoking status (%)**		
**Fomer**	12 (18.8)	2 (9.1)
**Current**	13 (20.3)	0
**None**	39 (60.9)	20 (90.1)
**ICS use (%)**	54 (84.4)	0
**Allergic history**	34 (52.3)	0
**Spirometry**		
**FEV1.0 (L/s)**	2.1 ± 0.73	2.36 ± 0.53
**%FEV1.0**	85.91 ± 21.42	94.76 ± 17.51
**%PEF**	84.3 ± 25.42	94.99 ± 14.84
**Sputum cell profile**		
**Total cell count** (x10^5^ cells/mL)	39.24 ± 37.13	34.28 ± 30.55
**Neutrophils %**	56.62 ± 24.62	61.83 ± 26.75
**Lymphocytes %**	2.73 ± 2.39	7.96 ± 7.41
**Macrophages %**	28.86 ± 22.19	29 ± 24.55
**Eosinophils %**	11.58 ± 16.66	2.72 ± 4.16
**White blood cells (cells/μL)**	6489 ± 1573	4899 ± 1058
**Blood eosinophils %**	5.56 ± 4.55	1.95 ± 0.57
**Blood IgE (IU/mL)**	460 ± 718	57.4 ± 42.2

Data are represented as mean ± sd or n (%), unless otherwise stated. HV, Healthy volunteer; ICS, inhaled corticosteroid; FVC, forced vital capacity; FEV1.0, forced expiratory volume in 1 s; PEF, peak expiratory flow

### Quantification of human LRG

The concentrations of sputum LRG were measured by sandwich enzyme-linked immunosorbent assay (ELISA). Monoclonal antibodies specific for human LRG (huLRB0091 and rbLRB0048) were used as previously described with minor modification [13]. For the measurement of sputum samples, we assessed the effect of DTT on the performance of ELISA, because this reductant was used for the processing of sputa and remained in the ELISA samples at the final concentration of 0.005%. In the presence of 0.005% DTT, the absorbance values of samples containing recombinant human LRG was decreased to 70% of those without DTT, but they still gave a linear relation with the added recombinant LRG. We therefore generated a standard curve using recombinant human LRG supplemented with 0.005% DTT to determine LRG levels of sputum samples.

### Mice

Animal experiments were approved by the Animal Research Committee of Kochi Medical School. Mice were maintained under specific pathogen free condition and physical condition was routinely monitored. All surgeries were performed under sodium pentobarbital anesthesia, and all efforts were made to minimize suffering. At the time of sacrifice, mice were euthanized by blood collection by inferior vena cava under the anesthesia, and organs were excised for further experiments. All animals were treated humanely, and experiments were conducted in accordance with institutional ethics guidelines.

### Murine model of asthma

To generate an asthma model in mice, C57BL/6 mice were sensitized on days 0 and 14 by intraperitoneal injection of 20 μg of ovalbumin (OVA) and 2 mg of Al(OH)_3_. Fourteen days after the second sensitization, mice were exposed to 1% OVA aerosols for 20 min daily for three consecutive days (days 28, 29 and 30) and control mice were exposed to aerosolized PBS. On day 32, BALF was obtained and LRG concentrations were determined by ELISA optimized for mouse samples.

### Collection of BALF

The mice were sacrificed with a lethal dose of pentobarbital, the tracheas were cannulated with a 20-gauge needle, and the lungs were lavaged once with 1 ml of saline and 0.05 mL of air. The lavage fluids were centrifuged for 10 min at 500 g, and the supernatants were collected. BALF samples were stored at -80˚C until measurement of LRG.

### Quantification of mouse LRG

BALF levels of mouse LRG were measured by the sandwich ELISA using specific antibodies. To generate monoclonal antibodies against LRG, rabbits were immunized with the recombinant mouse LRG protein. From rabbits, the genes of the variable regions of LRG-specific antibodies were cloned and inserted into an expression vector containing the constant region of mouse or rabbit IgG, as reported previously by Seeber et al [14] with a modification. Two clones (mLRA0010 and rLRA0094) were selected to construct a sandwich ELISA for the detection of mouse LRG. Briefly, 96-well microtiter plates were coated with the capture antibody (0.5 μg/ml mLRA0010) and blocked with 10 mM Tris-HCl, 150 mM NaCl, pH7.5, 0.01% Tween-20 (TBS-T) containing 0.5% BSA and Block Ace (DS Pharma Biomedical, Osaka, Japan). BALF were 20-fold diluted with blocking buffer before added to the plate and incubated for one hour. After washing, plates were incubated with the detection antibody (1 μg/ml rLRA0094), followed by peroxidase-conjugated anti-rabbit IgG (Southern Biotech). The standard curve was constructed by serial dilution of recombinant mouse LRG.

### Immunohistochemistry and PAS staining

Immunohistochemistry was performed using the ChemMate Envision method (DakoCytomation, Glostrup, Denmark). Briefly, four micro meter thick paraffin sections were de-waxed, rehydrated and incubated for 20 minutes in citrate buffer (10 mM citric acid, pH6.0) at 95°C—100°C for antigen retrieval. Sections were treated with 0.3% H_2_O_2_, then blocked with Blocking One (Nacalai, Kyoto, Japan) and incubated with anti-LRG1 monoclonal antibody (clone R322, IBL, Gunma, Japan) and anti-MUC5AC antibody (ab3649, abcam Cambridge, MA, USA) overnight at 4°C. After washing, sections were treated with Dako ChemMate ENVISION Kit (K5007) according to manufacturer’s instructions. All sections were counterstained with hematoxylin.

Serial sections from each mouse were stained with periodic acid-Schiff (PAS).

### Cell culture

Normal human bronchial epithelial cells (NHBE) (CC2540 Lot 0000429581, Lonza, Walkersville, MD) were maintained in bronchial epithelial cell growth medium (BEGM BulletKit, CC-3710, Lonza). Cells were cultured with or without 10 ng/mL IL-13 (213-ILB-005, R and D) for 5 days and further incubated with several cytokines for additional 24 h for western blot and 6 h for quantitative PCR. Cytokines used in this experiments were purchased from Peprotech (IL-6, TNF-α and IL-4) and R and D (IL-13, IL-25, IL-33 and TSLP).

### Western blot

Cell culture supernatants were collected and were subjected to SDS-PAGE and western blot analysis. Anti LRG1 antibody (HPA001888, Atlas Antibodies, Sweden) was used to detect LRG.

### Quantitative PCR analysis

Total RNA was isolated from cells and reverse-transcribed using the RNeasy Mini and QuantiTect Reverse Transcription Kits (QIAGEN, Tokyo, Japan), respectively. Real-time PCR (qPCR) was performed on ABI PRISM 7900HT Real-time system (Applied Biosystems, Darmstadt, Germany) using SYBR Premix Ex Taq (Takara Bio, Shiga, Japan). Target gene expression levels were normalized by glyceraldehyde-3-phosphate dehydrogenase (G3PDH) levels in each sample. Each reaction was performed in triplicate.

The primers for qPCR were designed and used as follows: human LRG, sense 5′- TTTACAGGTGAAACTCGGGG—3′, antisense 5′—ACCCCAAGCTAAGTGGGACT—3′; G3PDH, sense 5’- AGCAATGCCTCCTGCACCACCAAC—3’, 5’—CCGGAGGGGGCCATCCACAGTCT—3’; SPDEF, sense 5’-AGCCTACAGAAGGGCAGTGA—3’, antisense 5’-AACTCAGGGGTGCAGATGTC-3’; β-actin, sense 5’-AGCCTCGCCTTTGCCGA -3’, antisense 5’-CTGGTGCCTGGGGCG-3’

## Results and Discussion

In this study, we aimed to examine the potential of sputum LRG as a new biomarker for airway inflammation in asthma. To measure LRG concentrations in biological samples from human and mice, we used sandwich ELISA systems optimized for each species.

**Fig 1:**
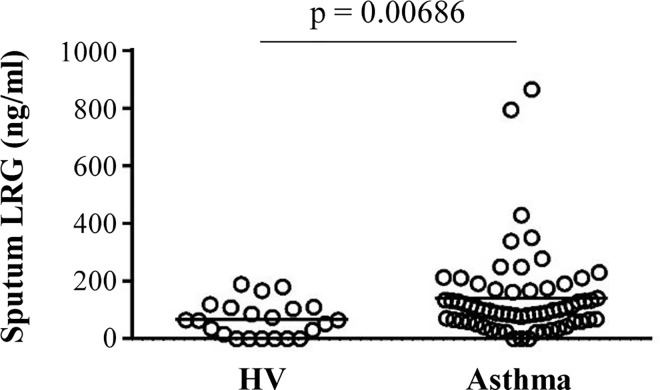
Up-regulation of LRG in asthmatic patients. Levels of LRG in sputum obtained from healthy volunteers and patients with asthma. Concentrations of sputum LRG were determined by ELISA. The Mann-Whitney U-test was used for statistical analysis. The individual values are provided in S2 File.

The detailed clinical characteristics of the study subjects are shown in Table 1. We obtained induced sputum samples from patients diagnosed with bronchial asthma (N = 64) and healthy volunteers without respiratory symptoms (N = 22). There was no significant difference in age or sex between the two groups. Sputum LRG levels were significantly higher in patients with asthma (140.5 ± 151.0 ng/ml) than in healthy volunteers (66.9 ± 59.6 ng/ml) (Fig 1). In asthmatic patients, disease duration of patients did not show a correlation with sputum LRG (Spearman rank correlation coefficients, p = 0.186). Sputum LRG levels were not significantly different between patients with or without smoking history (120.6 ± 80.4 or 153.2 ± 181.3, respectively. p = 0.962, Mann-Whitney U test), or allergic diathesis (156.9 ± 177.2 or 113.0 ± 85.3, respectively, p = 0.488, Mann-Whitney U test). In contrast, high levels of sputum LRG were observed in patients with inhaled corticosteroid treatment compared to those without this treatment (155.3 ± 158.3 or 60.3 ± 56.2, respectively, p = 0.009, Mann-Whitney U test). Statistical analysis of the correlation between sputum LRG levels and sputum cell profiles revealed that sputum LRG levels were most strongly associated with cellular infiltrates in the sputum (rs = 0.419, p = 0.0009; Table 2) and weakly with the percentages of sputum eosinophils (rs = 0.256, p = 0.042, Spearman rank correlation coefficients). In contrast, sputum LRG levels were not positively correlated with blood IgE and blood eosinophils, known biomarkers of allergic diseases (rs = 0.089, p = 0.625 and rs = -0.366 p = 0.045, respectively, by Spearman rank correlation coefficients). These results collectively suggest that sputum LRG is a potential biomarker of asthma, whose levels are not significantly affected by smoking or allergic history. Furthermore, the increase in sputum LRG is likely to reflect local airway inflammation related to various leucocytes, presumably not limited to eosinophilic inflammation. Our data also suggest that steroid treatment may affect LRG levels in the airway, but high LRG levels in patients treated with ICS may be explained by the general notion that this population contains patients with severe, unstable asthma compared to that without ICS treatment.

**Table 2:** Correlations between sputum LRG concentration and sputum total cell counts.

	Asthma	HV	Asthma + HV
**total cell count**	rs = 0.419	rs = 0.596	rs = 0.429
	p = 0.0009	p = 0.0066	p = 0.00008

**Fig 2:**
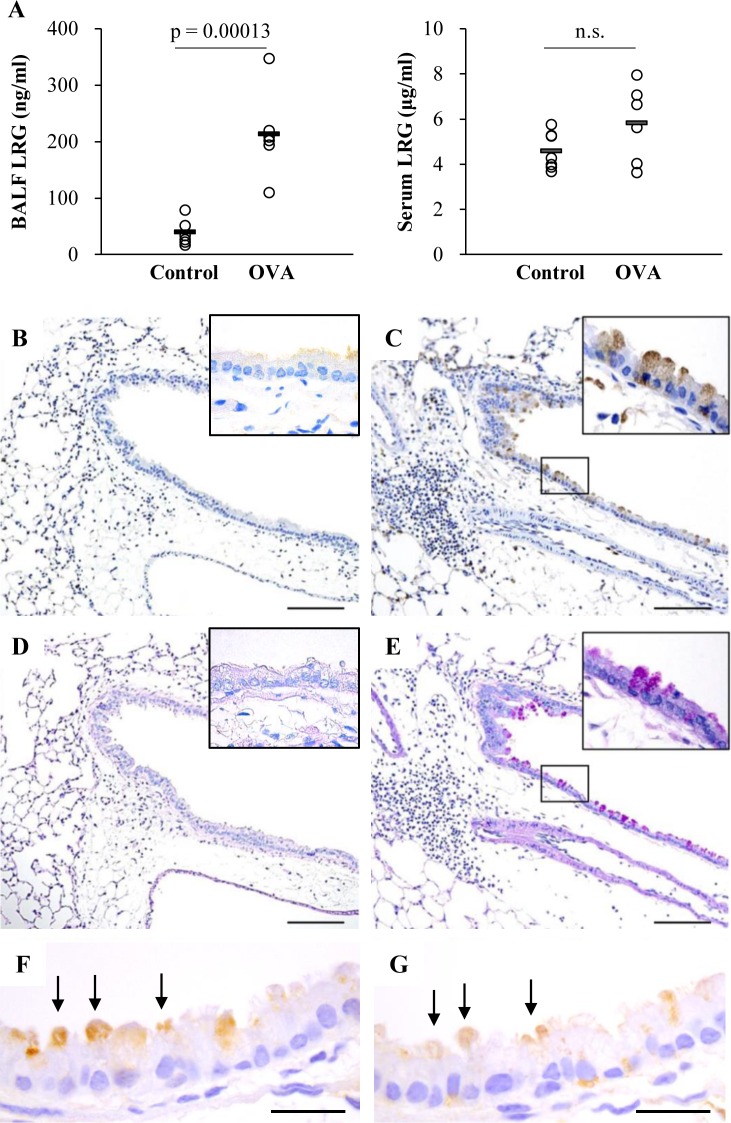
Detection of LRG in BALF, serum and lung section in a murine model of asthma. A) Levels of LRG in BALF and serum in a mouse model of asthma. Concentrations of BALF and serum LRG were determined by ELISA. The Student’s t-test was used for statistical analysis. The individual values are provided in S3B–S3E File) Localization of LRG in mouse lung. Paraffin sections of the lung from control (B and D) and OVA-treated (C and E) mouse were stained with anti-LRG antibody (B and C) and PAS (D and E). Scale bar, 100 μm. F and G) Immunohistochemisry of MUC5AC (F) and LRG (G) of the lung from OVA-treated mouse. Arrows show MUC5AC (F) or LRG (G) positive cells. Scale bar, 20 μm

Next, we investigated OVA-induced asthma model mice to determine the mechanism underlying the up-regulation of LRG in sputum. Consistent with the elevated sputum LRG levels in patients with asthma, LRG concentrations in the BALF were significantly higher in the mice treated with OVA (212.87 ± 69.96 ng/ml) than those in control mice (39.78 ± 19.98 ng/ml) (Fig 2A, left). While we had previously reported that serum LRG was increased in murine models of inflammatory diseases, such as DSS-induced colitis and LPS-induced sepsis [9], we observed no significant elevation in serum LRG of this model (control 4.57 ± 0.76 μg/ml, OVA 5.81 ± 1.57 μg/ml) (Fig 2A, right). During systemic inflammation, circulating cytokines such as IL-6, IL-1β and TNF-α can stimulate hepatocytes to release abundant LRG in sera [7]. However, in asthma model mice, we assume that LRG is produced locally at the inflammatory airways and is preferentially secreted into the airway.

To identify the cell types expressing LRG at the site of inflammation, paraffin sections of mouse lungs with or without OVA treatment were immunostained with anti-LRG antibody (Fig 2B and 2C). In control mice lungs, LRG was weakly detectable in a fraction of alveolar epithelial cells but not in bronchial epithelial cells. However, in OVA-treated mouse lungs, in which inflammatory cells accumulated around airways and blood vessels, a subpopulation of bronchial epithelial cells were intensely stained with anti-LRG antibody in addition to alveolar cells. The airway epithelium consists of two principal cell types: ciliated cells and non-ciliated secretory cells [15]. High-power microscopic imaging revealed that LRG was detected only in non-ciliated cells (S1A Fig). Furthermore, immunohistochemistry of Ezrin, the apical protein expressed in ciliated cells but not in non-ciliated cells, showed that LRG-positive cells corresponded to Ezrin-negative cells (S1B and S1C Fig). These results suggest that LRG is produced by non-ciliated secretory cells.

We then performed PAS staining using serial sections to specify mucus-producing epithelial cells (Fig 2D and 2E). When treated with OVA, PAS-stained bronchial epithelial cells markedly increased, implying that mucus production was enhanced by airway inflammation. Strikingly, LRG-stained bronchial epithelial cells in OVA-treated mice were associated with PAS-positive epithelial cells, indicating that LRG is especially up-regulated in mucus-producing cells. Furthermore, immunohistochemistry showed that the expression of LRG correlated with MUC5AC, a marker of goblet cell metaplasia in murine airways (Fig 2F and 2G) [16]. Given that LRG expression was increased in inflamed colonic mucosa [9], it is possible that mucosal epithelial cells are a critical source of LRG during inflammatory reactions. At present, whereas the role of LRG in the airway inflammation is unknown, locally secreted LRG may contribute to the pathogenesis of asthma, because LRG was reported to modulate signaling of TGF-β [17, 18], a major stimulator of subepithelial fibrosis and airway remodeling.

**Fig 3:**
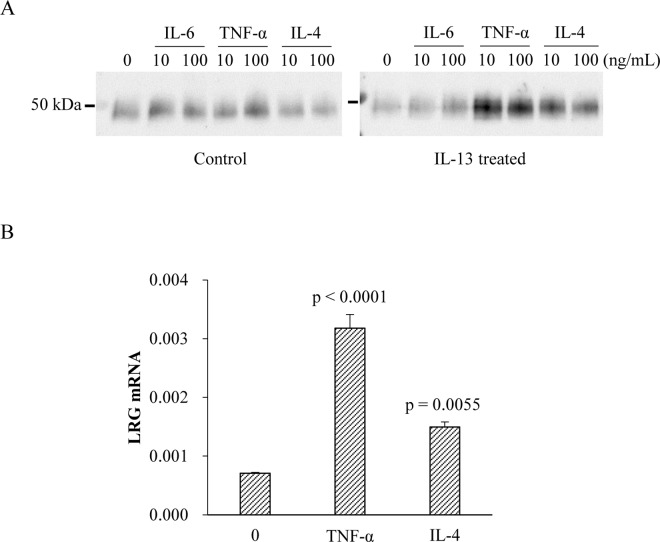
Induction of LRG in primary bronchial epithelial cells. A) LRG secretion in culture supernatant by primary bronchial epithelial cells. Cells were incubated with IL-13 for 5 days and then treated with indicated cytokines for 24 h. Control cells were incubated without IL-13 for 5 days and further stimulated by cytokines. LRG protein in culture supernatant was detected by western blot. B) LRG gene expression in primary bronchial epithelial cells treated with IL-13 for 5 days. Cells were stimulated by indicated cytokines for 6 h. LRG mRNA expression was analyzed by quantitative PCR. Dunnett’s test was used for statistical analysis. The individual values are provided in S4 File.

To investigate the mechanism of LRG induction in bronchial epithelial cells, we used normal human bronchial epithelial (NHBE) cells for analysis. In asthma, there is an increase in the number of goblet cells, referred to as goblet-cell metaplasia (GCM). GCM is thought to be due to the transdifferentiation of ciliated cells and Clara cells in bronchial epithelia, rather than due to the proliferation of goblet cells themselves [19]. Since IL-13 is known to induce GCM in bronchial epithelial cells *in vitro* and *in vivo* [20–23], aliquots of NHBE cells were pretreated with IL-13 [24]. As expected, the expression of SPDEF, a marker of GCM, was increased in IL-13-treated cells (S2A Fig). Western blot analysis revealed that NHBE cells, with or without IL-13 pretreatment, have an ability to secrete LRG in culture supernatants (Fig 3A). Cytokine stimulation of control cells cultured without IL-13 induced a marginal, if any, increase in LRG secretion (Fig 3A, left). In contrast, stimulation of IL-13-pretreated cells with TNF-α and IL-4 noticeably increased LRG in supernatants (Fig 3A, right). Furthermore, stimulation by these cytokines induced significant up-regulation of LRG gene expression in IL-13-pretreated cells (Fig 3B). This suggests that IL-13-induced transdifferentiation combined with stimulation by asthma-related cytokines such as TNF-α and IL-4 enables the epithelial cells to upregulate both transcription and secretion of LRG. This finding is in accordance with the results obtained in the murine asthma model, in which LRG production was observed in non-ciliated, mucus-producing cells in the airway. Thus, we speculate that transdifferentiation to mucus-producing cells is critical for epithelial cells to increase their LRG-producing ability, which is stimulated further by additional cytokines such as TNF-α and IL-4. In addition, because inflammatory cytokine signaling is known to be inhibited by corticosteroids, as demonstrated by the downregulation of TNF-α-induced VCAM or ICAM expression in bronchial epithelial cells [25, 26], our findings argue against the idea that ICS treatment directly increase LRG expression in the airways by enhancing proinflammatory signaling.

Recently, periostin has been highlighted as a potential target for the diagnosis of asthma [27, 28]. Like LRG, sputum periostin levels are elevated in patients with asthma [29] and airway epithelial cells were demonstrated to be the major source of periostin [30]. Interestingly, periostin is considered to be a biomarker of Th2-related eosinophilic inflammation and periostin gene expression is induced by IL-13 and IL-4 but not by TNF-α [30]. In contrast, whereas LRG was induced by IL-4 in IL-13-primed NHBE cells, IL-13 alone did not increase LRG levels in NHBE cells and TNF-α was the most potent in LRG induction (Fig 3). In addition, upon stimulation of transdifferentiated NHBE cells with TSLP, IL-25 and IL-33, Th2-promoting cytokines known as epithelial-related cytokines, LRG expression tended to be increased by IL-33 but not by TSLP and IL-25 (S2B Fig). It is suggested that IL-33 induced LRG gene expression via the proinflammatory pathway similar to IL-1β. Thus, LRG induction in epithelial cells is not specific to Th2-related condition, but rather mediated by several Th2 cytokines and prototypic inflammatory cytokines such as TNF-α. Because asthma is a complex syndrome and the disease can be driven not only by Th2 inflammation but also by non-Th2 immune response [31], sputum LRG may be useful to monitor both Th2 and non-Th2 airway inflammation in asthma.

In conclusion, we demonstrated that sputum LRG levels are significantly increased in patients with asthma. We also found that BALF LRG is increased in the murine asthma model and that LRG is expressed in asthmatic airway epithelia. Increased production and secretion of LRG in bronchial epithelial cells require not only the mucous differentiation of these cells but also the additional stimulation by the inflammatory cytokines such as TNF-α and IL-4. Our findings suggest that sputum LRG is a promising biomarker of local inflammation in asthmatic airways. It would be of great importance to examine sputum LRG levels before and after therapeutic intervention, in particular with ICS. Further prospective studies are underway to assess the clinical benefit of this novel biomarker.

## Supporting Information

S1 Fig **(A) Microscopy in high-power fields.** Microscopic observation of OVA treated mouse bronchi. A paraffin section of mouse lung was immunostained with anti-mouse LRG antibody. **(B) and (C) Localization of Ezrin and LRG in the lung.** Parrafin sections of the lung from OVA-treated mouse were stained with anti-Ezrin and anti-mouse LRG antibodies. Arrows show Ezrin-negative (B, dotted line) and LRG-positive (C) cells. Scale bar = 50 μm
(PDF)

S2 Fig Gene expressions of SPDEF and LRG in primary bronchial epithelial cells. (A) Change of SPDEF gene expression in primary bronchial epithelial cells. Cells were treated with or without 10 ng/mL of IL-13 for 5 days. SPDEF gene expression was measured by quantitative PCR. (B) LRG gene expression in cells treated with IL-13 was measured by quantitative PCR. The individual values are provided in S4 File.
(PDF)

S1 File Patients’ characteristics. Sheet 1. Result of DTT spiking experiments of human LRG ELISA. Sheet 2. Individual data sets of patients’ characteristics.
(XLSX)

S2 File Human LRG ELISA. Levels of LRG in sputum (Sheet 1) and serum (Sheet 2) of patients with asthma and healthy volunteers.
(XLSX)

S3 File Mouse LRG ELISA. Levels of LRG in BALF (Sheet 1) and serum (Sheet2) of OVA-treated or control mice.
(XLSX)

S4 File Gene expressions in bronchial epithelial cells. Expression of genes in primary bronchial cells measured by quantitative PCR. SPDEF (Sheet 1) and LRG (Sheet 2 and 3) mRNA was evaluated.
(XLSX)

## Abbreviations

BALF: bronchoalveolar lavage fluid
FeNO: fractional exhaled nitric oxide
LRG: leucine rich alpha-2 glycoprotein
ELISA: enzyme-linked immunosorbent assay
OVA: ovalbumin
PAS: periodic acid-Schiff
