# Supplementary figures and images for: Sputum Leucine-Rich Alpha-2 Glycoprotein as a Marker of Airway Inflammation in Asthma
Source: PLoS One. 2016 Sep 9;11(9):e0162672. doi: 10.1371/journal.pone.0162672 (PMC5017625; doi:10.1371/journal.pone.0162672)

S1 Fig

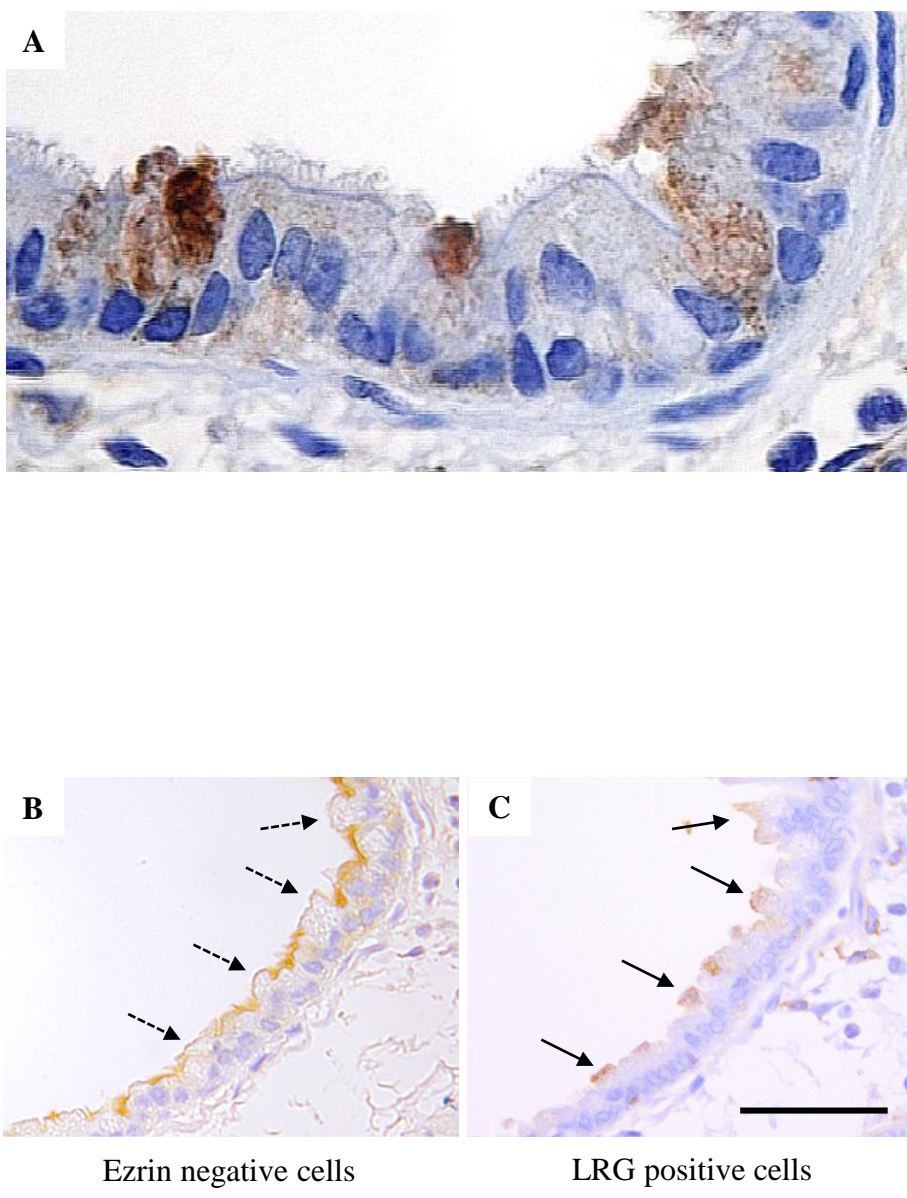

Supplement: S1 Fig — (A) Microscopy in high-power fields. Microscopic observation of OVA treated mouse bronchi. A paraffin section of mouse lung was immunostained with anti-mouse LRG antibody. (B) and (C) Localization of Ezrin and LRG in the lung. Parrafin sections of the lung from OVA-treated mouse were stained with anti-Ezrin and anti-mouse LRG antibodies. Arrows show Ezrin-negative (B, dotted line) and LRG-positive (C) cells. Scale bar = 50 μm (PDF) [file pone.0162672.s001.pdf]

S2 Fig

A

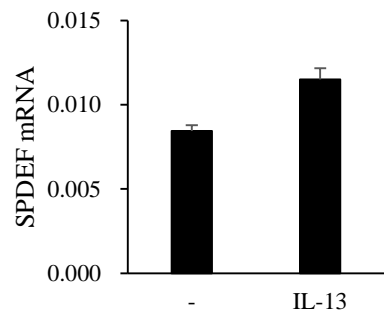

B

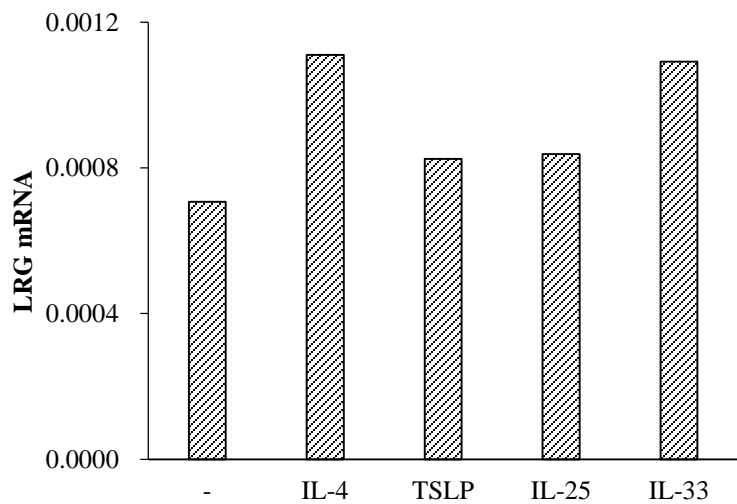

Supplement: S2 Fig — (A) Change of SPDEF gene expression in primary bronchial epithelial cells. Cells were treated with or without 10 ng/mL of IL-13 for 5 days. SPDEF gene expression was measured by quantitative PCR. (B) LRG gene expression in cells treated with IL-13 was measured by quantitative PCR. The individual values are provided in S4 File. (PDF) [file pone.0162672.s002.pdf]
